# Regional left ventricular myocardial T1 and velocity mapping: elevated extracellular volume fraction is associated with altered myocardial velocities

**DOI:** 10.1186/1532-429X-16-S1-P5

**Published:** 2014-01-16

**Authors:** Jeremy D Collins, Lewis Sommerville, Daniela Föll, Patrick Magrath, Bruce S Spottiswoode, Benjamin H Freed, James C Carr, Michael Markl

**Affiliations:** 1 Radiology, Northwestern University, Chicago, Illinois, USA; 2 Biomedical Engineering, Northwestern University, Chicago, Illinois, USA; 3 Cardiology, Northwestern University, Chicago, Illinois, USA; 4 Kardiologie und Angiologie, Universitäts Herzzentrum Freiburg, Freiberg, Germany; 5 Cardiovascular MR R&D, Siemens Healthcare, Chicago, Illinois, USA

## Background

Non-ischemic cardiomyopathy (NICM) is a common cause of left ventricular (LV) dysfunction and may be associated with myocardial fibrosis. Global LV systolic function may underestimate the impact of fibrosis on myocardial function and is insensitive to regional abnormalities in myocardial motion. Advances in cardiac MRI have enabled quantification of myocardial fibrosis through the calculation of the gadolinium extracellular volume fraction (Ve) using T1 mapping techniques employing the modified look-locker inversion recovery (MOLLI) correction. In addition, tissue phase mapping (TPM) enables assessment of regional myocardial velocities. The purpose of this study was to evaluate the impact of regional myocardial fibrosis on regional myocardial velocities. We hypothesize that regional scar will correlate with reduced regional systolic and diastolic velocities.

## Methods

CMR was performed in 38 patients (22 men, avg age 48.2 ± 18.6 yrs) with NICM on a 1.5T scanner (MAGNETOM Aera or Avanto, Siemens AG, Healthcare Sector, Erlangen, Germany). TPM was performed using a black-blood prepared cine phase-contrast sequence with tri-directional phase encoding in the short axis orientation (venc = 25 cm/sec, temp res = 24 msec, spatial res = 2.9 × 2.4 mm, thickness = 8 mm). T1 mapping was performed in identical slice positions using a MOLLI sequence comprised of three inversion pulses with single shot bSSFP diastolic readouts using a 3, 3, 5 schema with three recovery heart beats between inversion pulses (spatial res = 2.3 × 1.8 mm, thickness = 8 mm). T1 mapping images were acquired before and 10 to 25 minutes after 0.2 mmol/kg gadobenate dimeglumine (Multihance, Bracco Diagnostics, Monroe, NJ) injection. The Ve fraction was calculated as originally described by Jerosch-Harold. A reduced LV ejection fraction (EF) was defined as <50%. Analysis was based on a 16-segment AHA model.

## Results

**Figure 1:**
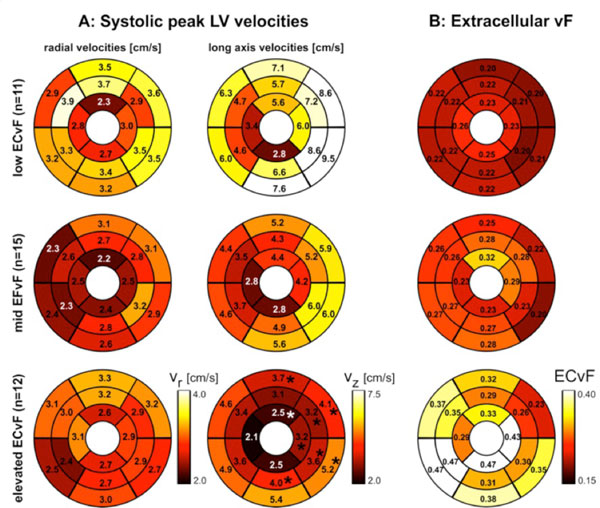
**A: Systolic left ventricular (LV) velocities in three patient subgroups with non-ischemic heart disease and normal extracellular volume fraction (Ve ≤ 0.25), borderline 0.25 < Ve < 0.3, and elevated Ve ≥ 0.3**. The individual plots show the distribution of peak systolic radial and long-axis myocardial velocities on the AHA 16-segment model. B: LV mapping of extracellular volume fraction (ECvF) derived from pre- and post-contrast T1-mapping. * Significant difference between patient groups after Bonferroni correction (p < 0.0031).

LVEF was reduced (31 ± 12%) in n = 12 and preserved (61 ± 6%) in n = 26 patients. The global Ve was similar for both reduced (0.28 ± 0.06) and preserved (0.29 ± 0.08) LVEF subgroups despite significant differences in LVEF (p < 0.001). Although average regional systolic velocities were reduced for the low LVEF cohort, significant differences were seen in only 1 segment for long-axis velocities and the distribution of regional Ve and diastolic peak velocities was similar between LVEF subgroups. Differences in regional peak velocities were most pronounced stratifying by normal (<25%), borderline (25-29%), or elevated (>30%) Ve (Figure 1). In patients with preserved LVEF, increasing Ve significantly correlated with reduced systolic (r = -0.21 and -0.35) and diastolic (r = 0.29 and 0.28) radial and longitudinal velocities respectively (p < 0.001).

## Conclusions

The association of elevated regional myocardial Ve with reduced myocardial velocities independent of LVEF suggests a closely interrelated regional tissue structure-function relationship in patients with NICM.

## Funding

None.

